# Does the brain sleep differently depending on intellectual abilities?

**DOI:** 10.1111/cns.14378

**Published:** 2023-07-23

**Authors:** Marine Thieux, Min Zhang, Anne Guignard‐Perret, Stéphanie Mazza, Sabine Plancoulaine, Aurore Guyon, Patricia Franco

**Affiliations:** ^1^ INSERM U1028, CNRS UMR5292 Lyon Neuroscience Research Center Lyon France; ^2^ Pediatric Sleep Unit, Department of Pediatric Clinical Epileptology, Sleep Disorders and Functional Neurology, Hôpital Femme Mère Enfant Hospices Civils de Lyon Lyon France; ^3^ Research on Healthcare Performance RESHAPE, INSERM U1290 Université Claude Bernard Lyon 1 Lyon France; ^4^ Inserm, INRAE, Center for Research in Epidemiology and Statistics (CRESS) Université Paris Cité and Université Sorbonne Paris Nord Paris France

**Keywords:** children, cognition, EEG, polysomnography, spectral analysis

## Abstract

**Aims:**

To compare the children's sleep electroencephalogram according to their intellectual profile.

**Methods:**

Children were grouped according to their Wechsler Intelligence Scale for Children (WISC) scores (17 with normal intelligence quotient [IQ, NIQ] and 24 with high IQ [HIQ]). Comparisons of spectral power between groups and its relationship with WISC scores were assessed using analyses of variance and linear regression models, adjusted for age and sex.

**Results:**

Children with HIQ had more rapid eye movement (REM) sleep, especially late at night, and more power in slow‐frequency bands during REM sleep than those with NIQ. There were also positive associations between the processing speed index and the spectral power in *β* bands in NREM sleep, and with the spectral power in *α*, *σ*, *β*, and *γ* bands in REM sleep, with different associations between groups.

**Conclusion:**

The enhanced power in slow bands during REM sleep in children with HIQ overlaps with that of typical REM sleep oscillations thought to be involved in emotional memory consolidation. The dissimilar relationships between spectral power and WISC scores in NIQ and HIQ groups may underlie functional differences in brain activity related to cognitive efficiency, questioning the direction of the relationship between sleep and cognitive functioning.

## Graphical abstract

The architecture of sleep in children varies with intelligence profiles. Compared to children with a normal intelligence quotient (IQ), children with high IQ have a higher REM sleep percentage during the third part of the night and more mean relative power in slow‐frequency bands during REM sleep.

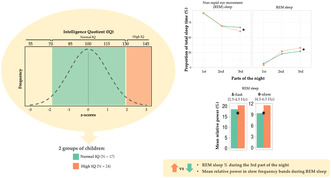

## 1 INTRODUCTION

Adequate sleep is a prerequisite for efficient daytime functioning. During childhood, sleep quality and quantity have been associated with academic, general, and specific cognitive performances such as memory and attention.^1^, ^2^, ^3^, ^4^ Although the mechanisms of these associations have been well theorized,^5^, ^6^, ^7^ the relationship between sleep and intellectual abilities in developing brains remains unclear.^8^

The sleep electroencephalogram (EEG) constitutes the visible part of the underlying brain anatomy and thus reflects the functional efficiency of neuronal networks in relation to maturational aspects.^9^ A wide range of studies have focused on non‐rapid eye movement (NREM) sleep spindles as intra‐ and inter‐individual differences in spindle activity are thought to reflect memory consolidation and cognitive abilities, respectively.^9^ After nearly two decades of research focusing on EEG markers of intellectual skills from childhood to adulthood, a recent meta‐analysis confirmed the modest positive association between general cognitive abilities and spindle amplitude (i.e., for both fast and slow spindles), without significant correlation for spindle duration or frequency, and ambiguous results for slow spindle density.^10^ Other sleep EEG oscillations, such as cycling alternating pattern (CAP), *θ*, and slow‐wave activity (SWA), have been associated with several cognitive domains during maturation.^11^, ^12^ In toddlers, *δ*‐slow and *θ*‐fast in various locations during NREM sleep (i.e., combining the sleep stages N2 and N3) were related to language, fine motor skills, and social skills,^13^ highlighting the multifactorial aspect of intelligence. Similarly, SWA development paralleled that of visuomotor skills in childhood^14^ and various skills depending on topographic parameters in subjects aged 2–26 years.^15^ In typically developing school‐aged children, positive associations have been found between CAP (i.e., CAP rate and A1 index) and fluid reasoning abilities, when controlling for age,^16^ and between the spectral power in the *α*‐, *σ*‐, and, *β*‐frequency bands in NREM sleep, especially in central and parietal regions, and subscales of the Wechsler Intelligence Scales for Children (WISC) (i.e., intelligence quotient (IQ), fluid IQ, and working memory index).^17^, ^18^

Surprisingly, although the aforementioned studies have shown promising results, only a few studies have examined sleep, especially its EEG spectral components, in children with high cognitive abilities. Early studies, conducted in small samples, have reported conflicting results. One study has suggested that, compared to controls with an IQ in the normal range (normal IQ, NIQ), children with a high IQ (HIQ) had a longer total sleep time (TST) and NREM sleep and lower eye movement density during rapid eye movements (REM) sleep (REM density). A negative correlation between REM density and IQ (i.e., total and verbal IQ) was also reported.^19^ Another study suggested that children with HIQ had more REM sleep with higher REM density.^20^ More recently, as reported in adolescents,^21^ our team showed that children and adolescents with HIQ had more REM sleep compared to those with NIQ.^22^

To bridge this gap, the main objective of the present study was to compare the sleep EEG power density spectra between children with HIQ and NIQ. Since intelligence is considered a stable construct and sleep EEG spectrum a neurophysiological fingerprint,^9^, ^23^ they can be considered as two facets of the same individual trait.^17^ For this reason, we hypothesized that: (i) children with HIQ and those with NIQ would differ in terms of sleep EEG power density spectra; (ii) these differences would be prominent in REM sleep; and (iii) EEG power density spectra will be associated with different IQ components.

## 2 METHODS

### 2.1 Participants

Forty‐four children were included in this retrospective case–control study. Inclusion criteria were as follows: (i) age ≥6 and ≤16 years old, (ii) no medical disease, (iii) no psychiatric disorder, (iv) no autism spectrum disorder, and (v) medication free at the time of the examination. Children were recruited from the ENSOM study (No. 2015‐A00703‐46, NCT02785328) and from the department of developmental psychology of the Lyon University Hospital, France, where they consulted mainly for school orientation issues, between 2016 and 2018. This retrospective study was conducted using medical records. All patients were informed of the study and had the opportunity to refuse the use of their medical data. All experimental procedures were carried out in accordance with the Declaration of Helsinki.

### 2.2 Procedure

All children underwent a systematic interview with a pediatric sleep specialist and a pediatric psychiatrist, a psychometric assessment, and a one‐night polysomnography. The EEG power spectral analysis was computed afterward.

### 2.3 Psychometric assessment

The psychometric assessments were performed by experienced psychologists using the French version of the WISC,^24^ providing the IQ which is the combination of the Verbal Comprehension Index (VCI), Perceptual Reasoning Index (PRI), Working Memory Index (WMI), and Processing Speed Index (PSI). Each index is normalized within each age group (mean 100, standard deviation 15). When the IQ was in the area of high intellectual potential^25^, ^26^ (i.e., VCI, PRI, or IQ ≥130, representing more than 2 standard deviations from the mean of the normal distribution), children were included in the HIQ group, otherwise, children were included in the NIQ group. An absolute difference ≥15 between VCI and PRI is known as significant verbal performance discrepancy (SVPD) and highlights the heterogeneity of the cognitive profile. In this case, the IQ was not calculated and an alternative score was computed from the VCI and PRI alone. This score is supposed to provide a better estimate of the general cognitive functioning (i.e., the *g* factor) and to be less sensitive to age‐related changes or neuropsychological damage.^27^

Three validated questionnaires were also used: the Adapted Epworth Sleepiness Scale (AESS),^28^ the Insomnia Severity Index (ISI),^29^ and the Child Depression Inventory (CDI),^30^ to assess sleepiness, insomnia, and depressive symptomatology, respectively.

### 2.4 Polysomnography

Single‐night ambulatory polysomnography (PSG) was conducted with a portative sleep recording system (DREAM, Medatec). The PSG included eight EEG leads referenced to the mastoids according to the 10–20 system (F1‐A2, F2‐A1, C3‐A2, C4‐A1, T3‐A2, T4‐A1, O1‐A2, and O2‐A1), right and left electrooculograms, electromyography on the levator menti surface and left and right anterior tibialis muscle, thoracic and abdominal respiratory belts, and an electrocardiogram. The EEG signal was sampled at 256 Hz; impedances were kept below 10 kΩ. Sleep was scored visually by an experienced specialist according to the pediatric criteria of the American Academy of Sleep Medicine.^31^ TST, sleep efficiency, sleep and REM latency, duration and percentage of stages N1, N2, and N3, NREM (i.e., N1 + N2 + N3) and REM sleep, wake after sleep onset (WASO), arousal index, and number and duration of sleep cycles were collected.

### 2.5 Spectral analysis

Spectral analysis was conducted using PRANA (PhiTools). A pre‐treatment before the analysis was performed by an automatic rejection of the epochs containing eye and body movements, muscular activity, sweating, and electrode artifacts, and was followed by a visual inspection. Three children were excluded because of the poor signal quality. Epochs with less than 50% of artifacts were then analyzed to obtain spectral data in each sleep stage. The study was conducted on the C3‐A2 signal. Absolute EEG power spectra (μV^2^/Hz) were computed using the fast Fourier transform (FFT) method using 4 s Hanning‐tapered windows with a 50% overlap between consecutive epochs (i.e., 0.25 Hz resolution). The power spectrum was then divided into frequency bands: delta (*δ*‐slow: 0.75–2.5 Hz and fast: 2.5–4.5 Hz), theta (*θ*‐slow: 4.5–6.5 Hz and fast: 6.5–8.5 Hz), alpha (*α*‐slow: 8.5–10.5 Hz and fast: 10.5–12.5 Hz), sigma (*σ*: 12.5–15.5 Hz), beta (*β*‐slow: 15.5–22.5 Hz and fast: 22.5–35.5 Hz), and gamma (γ: 35.5‐45 Hz) and averaged over 30‐s epochs. The relative power density (%) was calculated by dividing the absolute power of each frequency band by the sum of the power of all the frequency bands. The average value for each frequency band was calculated for each subject in NREM and REM. Knowing that, under physiological conditions, slow‐wave sleep (i.e., N3) decreases exponentially across the night while REM sleep increases, the night was also divided into three equal parts for each subject and the average value for each frequency band was computed in each part for both REM and NREM sleep. During REM sleep, automatic detection of rapid eye movements followed by a visual inspection to reject REM related to micro‐arousals was conducted to compute REM density.

### 2.6 Statistical analysis

Statistical analysis was conducted using R (version 4.1.2).^32^ The statistical significance was set to a *p*‐value below 0.05 for all tests and effect sizes were calculated from standard eta squared (*η* ^2^). Missing data were excluded from the analysis.

First, to compare age, sex, WISC index, questionnaires, and sleep macrostructure between groups (HIQ vs. NIQ), Fisher's exact test for dichotomous variables and Wilcoxon or *t*‐test for continuous variables were computed according to the results of the Shapiro–Wilk tests. Age and sex comparisons between the two recruitment methods were also computed using the same methods.

Then, type II variance analyses (R‐car package) were applied to multivariate models (R‐stats package). For these analyses, the variables to be explained were the mean relative power, REM density, and *δ* absolute power (mean and total) in NREM sleep. A logarithmic transformation was applied to power values in each frequency band. The first analysis focused on the group as the explanatory variable, adjusted for age and sex. A similar method was used to assess the effect of the group on each part of the night. Finally, linear regression models were used, with the same variables to be explained. This analysis focused on WISC characteristics, taking each index (i.e., VCI, PRI, WMI, and, PSI) as the explanatory variable, accounting for age, sex, and group. Age and sex were included because of their influence on sleep EEG parameters during typical development.^12^, ^33^, ^34^, ^35^, ^36^, ^37^

## 3 RESULTS

### 3.1 Participants' characteristics and sleep macrostructure

Children were grouped according to the IQ assessment: 17 with NIQ and 24 with HIQ. There was no significant difference between groups in terms of age and sex. Children with HIQ were recruited mainly in the department of developmental psychology of the Lyon University Hospital (88%), whereas those with NIQ were recruited mostly from the ENSOM study (88%). There was no significant difference between the recruitment method in terms of age and sex (*p* > 0.05). Compared to children with NIQ, children with HIQ had a higher IQ, VCI, PRI, and WMI and a higher proportion of SVPD. There was no significant difference between groups for the PSI (Table 1) or the questionnaire results (Table S1). Regarding sleep macrostructure, there was no significant difference between groups except that children with HIQ had a higher proportion of REM sleep than children with NIQ (23% vs. 20%, *p* = 0.03; Table S2).

**TABLE 1:** Demographic and WISC characteristics of children with HIQ and NIQ.

	NIQ	*n*	HIQ	*n*	*p*
Sex, boys, *n* (%)	12 (71)	17	16 (67)	24	1.00
Age	9.1 (7.3–15.1)	17	11.3 (7–13.8)	24	0.28
VCI	114 (82–128)	17	143 (123–155)	24	<0.001
PRI	104 (77–124)	17	126 (96–148)	24	<0.001
WMI	102 (60–124)	16	115 (85–133)	23	0.01
PSI	103 (64–131)	16	106 (86–143)	22	0.29
IQ	111 (72–127)	16	131 (115–147)	22	<0.001
SVPD, *n* (%)	4 (24)	17	15 (63)	24	0.03

*Note*: All values in columns 2 and 4 are reported as median (range) and *n* (%) when specified. The values in columns 3 and 5 indicate the sample size. The last column indicates *p*‐value for comparisons between the two groups (NIQ vs. HIQ) using Fisher's Exact test, *t*‐test or Wilcoxon test according to the results of the Shapiro–Wilk tests.

Abbreviations: HIQ, high intellectual quotient; IQ, intelligence quotient; NIQ, normal intellectual quotient; PRI, perceptual reasoning index; PSI, processing speed index; SVPD, significant verbal performance discrepancy; VCI, verbal comprehension index; WISC, Wechsler intelligence scale for children; WMI, working memory index.

**FIGURE 1:**
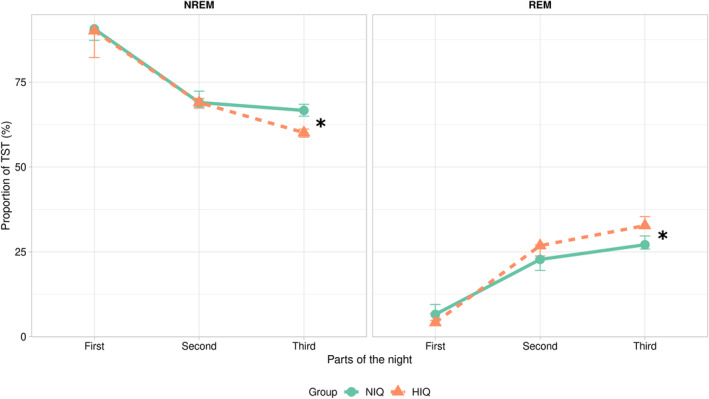
Across‐night sleep dynamics. Proportion (/total sleep time, TST) of non‐rapid eye movements (NREM) and REM sleep in each part of the night in children with a normal intelligence quotient (NIQ) (green) and a high IQ (HIQ) (orange) (R‐ggplot2).

When assessing the sleep dynamics across the night, there was no significant difference between HIQ and NIQ regarding the proportion of NREM (first: 90% vs. 91%, *p* = 0.67; second: 69% vs. 69%, *p* = 0.64) and REM sleep (first: 4% vs. 7%, *p* = 0.13, second: 27% vs. 23%, *p* = 0.09) during the first and second parts of the night. For the third part, children with HIQ had significantly less NREM (60% vs. 67%, *p* < 0.01) and significantly more REM sleep (30% vs. 19%, *p* < 0.01) than children with NIQ (Figure 1).

### 3.2 Sleep EEG power spectra in children with HIQ and NIQ

#### 3.2.1 Comparisons for NREM sleep

**FIGURE 2:**
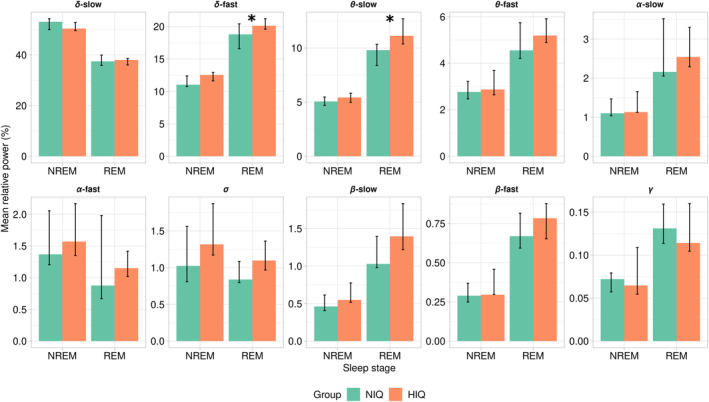
Power spectra between groups. Mean relative spectral power (%) in each frequency band in non‐rapid eye movements (NREM) and REM sleep, in children with normal intelligence quotient (NIQ) (green) and high IQ (HIQ) (orange) unadjusted (R‐ggplot2). Significant differences are reported with a star: *p < 0.05.

There was no significant difference between groups for the mean relative power in each frequency band (Figure 2) or for the absolute power (mean and total) in the *δ*‐frequency bands in NREM, after adjusting for age and sex (Table S3).

#### 3.2.2 Comparisons for REM sleep

In adjusted analyses on age and sex, there were significant differences between groups for the mean relative power in the *δ*‐fast‐ and *θ*‐slow‐frequency bands which were higher in children with HIQ compared to those with NIQ (Figure 2; Table 2). When looking at the REM sleep dynamics across the night, the mean relative power in the *δ*‐fast‐frequency band was higher in children with HIQ compared to children with NIQ in the first and second parts of the night (part 1: 21 vs. 20, *p* = 0.03, *η* ^2^ = 0.167; part 2: 20.8 vs. 19.1, *p* = 0.01, *η* ^2^ = 0.167) and a similar tendency for the third part (19.7 vs. 17.8, *p* = 0.06, *η* ^2^ = 0.119). The mean relative power in the *θ*‐slow‐frequency band during REM sleep was higher in children with HIQ compared to children with NIQ in every part of the night (part 1: 9.7 vs. 8.3, *p* < 0.01, *η* ^2^ = 0.239; part 2: 10.3 vs. 9.1, *p* = 0.01, *η* ^2^ = 0.174; and part 3: 12.1 vs. 10.4, *p* = 0.02, *η* ^2^ = 0.207). There was no other difference between groups during REM sleep.

**TABLE 2:** EEG spectral characteristics of children with HIQ and NIQ during REM sleep.

	NIQ	HIQ	*p* ^unadjusted^	*p*	*η* ^2^
Relative power					
*δ*‐slow	37.5 (28.2–48.9)	38 (31.1–42.2)	0.67	0.89	0.109
*δ*‐fast	18.8 (12–26.7)	20.1 (17.5–25.3)	0.02	0.02	0.158
*θ*‐slow	9.8 (4.8–12)	11.1 (8–21.2)	0.01	<0.01	0.211
*θ*‐fast	4.6 (2.7–8.4)	5.2 (3.6–7.7)	0.23	0.37	0.147
*α*‐slow	2.2 (1.3–6.5)	2.5 (1.3–5.5)	0.87	0.74	0.187
*α*‐fast	0.9 (0.7–6.1)	1.2 (0.7–2.4)	0.74	0.82	0.262
*σ*	0.8 (0.6–1.6)	1.1 (0.6–2.6)	0.10	0.21	0.346
*β*‐slow	1 (0.7–2)	1.4 (0.6–3.7)	0.09	0.16	0.314
*β*‐fast	0.7 (0.4–1.2)	0.8 (0.4–1.4)	0.54	0.69	0.084
*γ*	0.1 (0.1–0.2)	0.1 (0.1–0.3)	0.58	0.45	0.045
Eye movements					
REM density, *n*/min	3.3 (0.3–12.7)	3.8 (0.5–8.8)	0.58	0.78	0.082

*Note*: All values in columns 2 and 3 are reported as median (range), and *p*‐values are reported for unadjusted (*p*‐unadjusted) and adjusted (*p*) models in columns 4 and 5 respectively. Analyses were conducted on log‐transformed values; *η* ^2^ stands for the effect size of the model adjusted for age and sex (column 6).

Abbreviations: HIQ, high intellectual quotient; NIQ, normal intellectual quotient; REM, rapid eye movement.

### 3.3 Relationship between WISC characteristics and sleep EEG power spectra

In NREM sleep, when adjusting for age, sex, and group, the PSI was associated with the mean relative power in the *β*‐slow‐frequency band (*F* = 4.56, *p* = 0.04, *η* ^2^ = 0.319): the higher the PSI, the higher *β*‐slow mean relative power. There was a tendency for this association in children with HIQ (*F* = 3.48, *p* = 0.08, *η* ^2^ = 0.369) but not in children with NIQ (*F* = 1.63, *p* = 0.23, *η* ^2^ = 0.237). There was no association between WISC scores and the mean relative power in the other frequency bands during NREM sleep.

**FIGURE 3:**
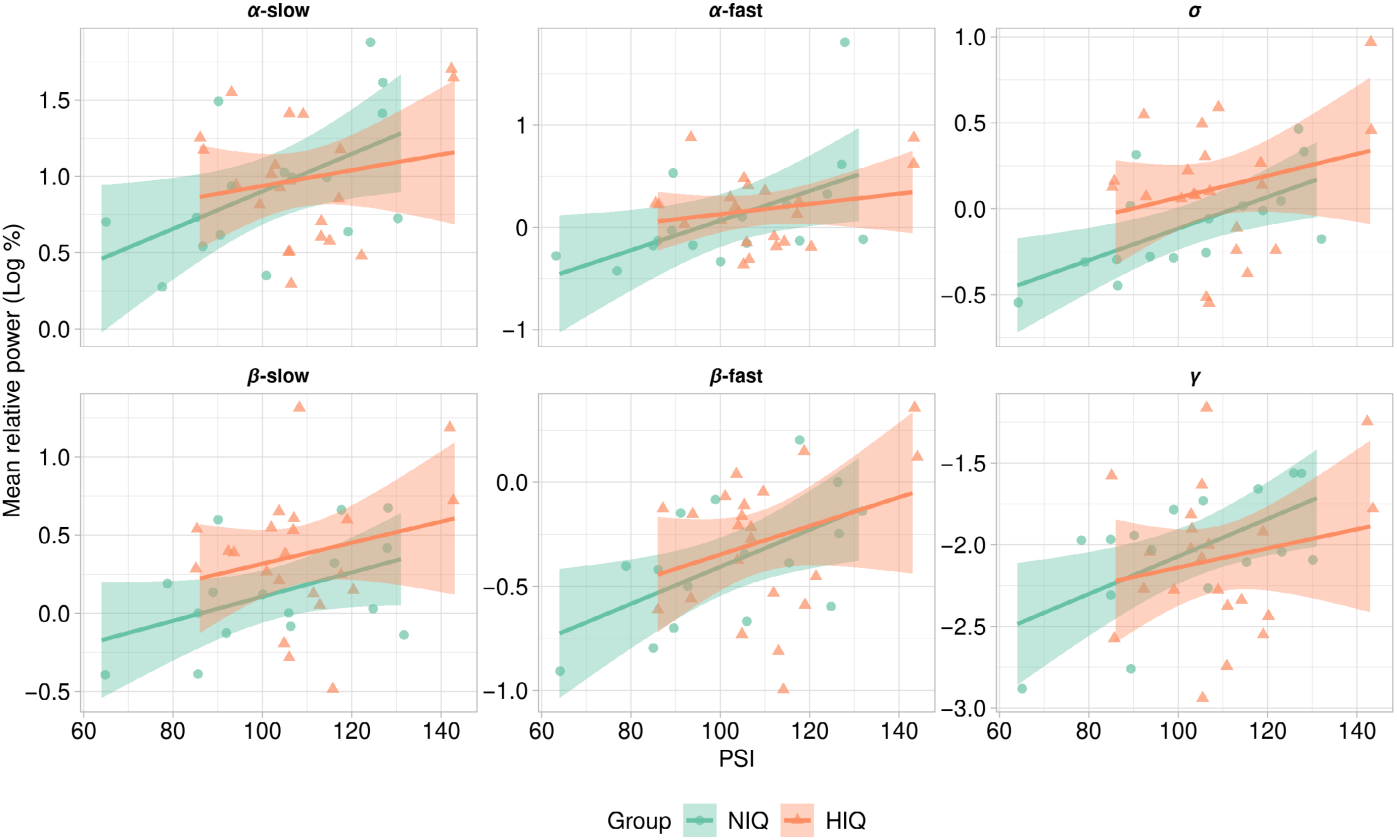
Associations between mean relative power in REM sleep and PSI. Each point represents the logarithmic transformation of the mean relative spectral power (%) according to the processing speed index (PSI) of the Wechsler Intelligence Scale for Children (WISC) in each frequency band in rapid eye movements (REM) sleep for children with normal intelligence quotient (NIQ) (green) and high IQ (HIQ) (orange) (R‐ggplot2).

In REM sleep, when adjusting for age, sex, and group, there were positive associations between the PSI and the mean relative power in the *α*‐slow, *α*‐fast, *σ*, *β*‐slow, *β*‐fast, and γ‐frequency bands (Table 3): the higher the PSI, the higher the mean relative power in these bands (Figure 3). In the NIQ group, the PSI was positively associated, or at least tended to be, with the mean relative power in all these frequency bands. In the HIQ group, the PSI was only associated with the mean relative power in the *σ*‐frequency band, and a tendency was observed with the *β*‐slow‐frequency band (Table 3). There was no other association between WISC scores and the mean relative power in the other frequency bands during REM sleep.

**TABLE 3:** Associations between relative sleep EEG frequency bands (log‐transformed) during REM sleep and the PSI in the entire group of children and the NIQ and HIQ groups.

Band	Entire group			NIQ			HIQ		
	*F*	*η* ^2^	*p*	*F*	*η* ^2^	*p*	*F*	*η* ^2^	*p*
*α*‐slow	5.72	0.412	0.02*	3.87	0.387	0.07#	1.80	0.506	0.20
*α*‐fast	9.60	0.516	<0.01*	5.28	0.501	0.04*	2.51	0.589	0.13
*σ*	10.95	0.619	<0.01*	12.10	0.723	<0.01*	4.27	0.572	0.05*
*β*‐slow	7.41	0.515	0.01*	4.18	0.562	0.06#	4.12	0.451	0.06#
*β*‐fast	8.85	0.324	0.01*	6.70	0.470	0.02*	2.85	0.220	0.11
*γ*	4.29	0.196	0.05*	7.25	0.455	0.02*	0.35	0.106	0.56

*Note*: All values in columns 3, 4, and 5 refer to the associations for the entire group; values in columns 6, 7, and 8 refer to the associations for the NIQ group; meanwhile, values in the last three columns refer to the associations for the HIQ group. *F* stands for Fisher's *F* test coefficient, *η* ^2^ stands for the effect size of the model adjusted for age and sex (and group for the “entire group” analysis), and *p* stands for the *p*‐value. Significant associations (*p* ≤ 0.05) are reported with a * and trends (*p* ≤ 0.10) are reported with a #.

Abbreviations: HIQ, high intellectual quotient; NIQ, normal intellectual quotient; PSI, processing speed index; REM, rapid eye movement.

## 4 DISCUSSION

While reaffirming the relationship between sleep and cognition, the present study also suggests that sleep microstructure aspects other than sleep spindles vary with intellectual abilities.

### 4.1 Children with HIQ exhibit more *δ*‐fast and *θ*‐slow mean relative power during REM sleep

According to our first and second hypotheses, children with HIQ differ from those with NIQ in terms of certain spectral characteristics, and these differences are prominent in REM sleep. Throughout the night, children with HIQ had more mean relative power in the *δ*‐fast and *θ*‐slow‐frequency bands during REM sleep than children with NIQ. Theta activity in REM sleep has been associated with the emotional aspect of memory consolidation, in both adults and children.^38^, ^39^, ^40^ Interestingly, the range of the frequency bands for which the mean relative power was increased in children with HIQ coincides with the frequency of typical REM sleep oscillations. Sawtooth waves (STWs) are generally described as bursts of medium‐amplitude, triangular‐shaped waves, between 2 and 6 Hz, with maximum amplitude in the frontocentral regions.^31^, ^41^, ^42^, ^43^ They usually precede or accompany the onset of REM sleep; often increase in density before/during REM may reflect the transition between tonic and phasic REM sleep and are more frequent late at night.^42^, ^43^, ^44^, ^45^, ^46^ Little is known about the mechanisms by which they are generated and their functional role. Their occurrence has been associated with an in‐phase increase in fast‐frequency activity, suggesting an activating effect.^47^ In a recent study evaluating the intracranial correlates of scalp STW using stereo‐EEG‐PSG, STWs were associated with a generalized increase in 2–4 Hz power (i.e., except in the occipital cortex) and were concomitant with fast‐frequency activities, including ripples. By driving fast activities, STW could be involved in the synchronized reactivations that underlie REM sleep memory consolidation, and more specifically with an emotional component.^45^ It has been proposed that STW may be related to ponto‐geniculo‐occipital oscillations because of their common characteristics,^43^, ^44^, ^47^ and that STWs may be understood as cortical correlates of ascending activation associated with phasic events, reflecting a widespread activation state.^45^ Thus, along with the increased activation of thalamocortical networks that underlie sleep spindles in higher‐performing individuals,^48^ a trait‐like neurological specificity for STW generation could be suggested in individuals with HIQ. As has been suggested for NREM sleep spindle activity,^9^ one could hypothesize that a possible night‐to‐night difference in REM sleep *δ*/*θ* activity could predict memory consolidation while inter‐individual differences could forecast intellectual abilities.

### 4.2 Associations between processing speed and EEG power spectra

Rather in line with our third hypothesis, the PSI was positively associated with the mean relative power in the *α*‐, *σ*‐, *β*‐, and γ‐frequency bands during REM sleep and in the *β*‐slow‐frequency band during NREM sleep, after adjusting for age and sex. In children, the EEG absolute power in *α*‐, *σ*‐, and *β*‐frequency bands in the central regions during NREM sleep has been positively correlated with the IQ, PRI, and WMI, as was the REM sleep although details were not provided.^17^ In another study conducted in adults, after adjusting for age, the relative power in the anterior regions during REM sleep was associated with a measure of fluid intelligence (i.e., a measure of fluid intelligence, close to the PRI).^49^ This association was negative for the power in the 2.25–5.25 Hz frequency range and positive for the 10.25–26.75 Hz frequency range, with sexual dimorphism. Together with the present study, these observations suggest that EEG frequencies in the ranges of 8.5–45 Hz are somehow related to intellectual abilities.

### 4.3 EEG spectral power according to intelligence: Differential patterns of associations

The study of the relationships between PSI and EEG spectral power showed different patterns according to the group. The associations observed during REM sleep were mainly found in children with NIQ, while in NREM sleep, the association appeared to be driven by the HIQ group. The lower dispersion of PSI scores among children with HIQ might explain these discrepancies.

Taken together, the differential association according to the IQ group between EEG activities and PSI as well as the specificities in sleep macro‐ and micro‐structure might underline functional differences in brain activity between both groups. First, it is believed that individuals with high cognitive efficiency generate a lower cortical activation and require fewer brain regions to cope with a task.^50^ Magnetic resonance imaging data have shown an enhanced intra‐ and interhemispheric white matter integrity,^25^ in addition to less segregation, less modularization, and a more global integration regarding brain network associations in children with HIQ compared to controls.^51^ Second, in line with the present results, high cognitive abilities have previously been related to fast frequencies during NREM sleep, without any association with slow frequencies.^17^ This could suggest less cognitive demand during wakefulness or a more efficient recuperation, characterized by a diminished need for slow activity during NREM sleep in children with high cognitive abilities. Supporting this idea, the shorter sleep duration classically found in this population has been explained in terms of a more efficient neuronal recovery.^8^ This short sleeper profile was not found in the present study, but the results herein regarding sleep macrostructure may support these observations. Compared to NIQ, children with HIQ have more REM sleep and this difference is mainly based on the third part of the night, where children with HIQ trade NREM for REM sleep. From another perspective, this difference could also be understood as a higher need for REM sleep. It has been proposed that the intellectual abilities of children with HIQ reflect a faster development that does not apply to affective domains, leading to a “developmental asynchrony,” with a pervasive impact on daily life.^52^, ^53^ The dysregulation of emotional processing in children with HIQ might lead to a higher need for emotional regulation, which could be achieved during REM sleep, as the latter enables the strengthening of emotional memories and the progressive fading of their affective tone.^54^, ^55^ These hypotheses must be further investigated.

### 4.4 Limitations and perspectives

This study has some limitations. To begin with, solely one‐night PSG was recorded, which may not reflect usual sleep characteristics and does not allow control of the time awake. However, children were sleeping at home and had a median total sleep time of almost 9 h with good efficiency (i.e., >95%). Also, since there was no significant difference in the *δ* absolute power during NREM sleep across the night between both groups, this suggests that the other results were not driven by sleep deprivation or time spent awake. Moreover, while the analyses considered age and sex, no adjustment was made for puberty, which is known to influence sleep EEG^56^; future studies may consider Tanner stages. In addition, WISC scores were used as a proxy for intellectual abilities. Each index is a combination of subtests, themselves involving different cognitive processes, which may have led to overlooking potential relationships between intelligence specificities and sleep EEG parameters. In this perspective, a previous study also found a relationship between spindle density and the PSI, without association with the IQ.^57^ The PSI might represent the purest measure compared to other WISC indexes, highlighting the need to examine specific cognitive subcomponents. Furthermore, the EEG power spectrum was only computed from the C3‐A2 signal. Other regional changes need to be evaluated, in line with the theory of local regulation of vigilance states, which suggests that regional changes in the sleep EEG may be use dependent.^58^ Finally, although the study was conducted using a rigorous methodology (i.e., visual rejection of artifacts and spectral analysis in multiple‐frequency bands calculated for NREM and REM sleep), the size of the dataset provides limited statistical power, and the results need to be replicated to consolidate our understanding of the relationship between sleep EEG and intellectual abilities. Future studies could use a longitudinal design to address the maturation effect and specific cognitive tasks, taking into account topographic parameters.^12^, ^18^

## 5 CONCLUSIONS

In this study, children with HIQ had more REM sleep, especially during the latter part of the night, with an increase in the mean relative power of the *δ*‐fast‐ and *θ*‐slow‐frequency bands corresponding to the frequency of typical REM sleep oscillations (i.e., STW). This study reinforces the importance of REM sleep in various aspects of cognition^54^, ^59^, ^60^ and questions the significance of STW in the genesis and functional role of REM sleep. The differences in associations between the PSI and the sleep frequency bands between groups suggest the existence of an alternative functioning in those with HIQ, particularly during REM sleep. This raises questions about the direction of the relationship between sleep and intellectual efficiency and, about the mechanisms underlying these sleep particularities. To conclude, this study reaffirms that, apart from sleep spindles, there is a wide range of relevant biomarkers of intellectual abilities that must be studied in the developing sleeping brain.

## AUTHOR CONTRIBUTIONS

Aurore Guyon, Patricia Franco, and Marine Thieux conceived and designed the study; Anne Guignard‐Perret acquired the data; Marine Thieux, Min Zhang and Sabine Plancoulaine analyzed the data; Aurore Guyon, Patricia Franco, and Marine Thieux interpreted the data and wrote the first draft of the manuscript; Aurore Guyon, Anne Guignard‐Perret, Marine Thieux, Min Zhang, Patricia Franco, Stéphanie Mazza, and Sabine Plancoulaine reviewed and corrected the final version the manuscript. All authors contributed to the article and approved the submitted version.

## FUNDING INFORMATION

This work was supported by the French National Agency for Research (ANR, grant number ANR‐15‐CE33‐0003), MT was supported by a French Grant from the SFRMS, and MZ was supported by a Chinese Grant from “China Scholarship Concil.”

## CONFLICT OF INTEREST STATEMENT

The authors declare no competing financial interests.

## Supporting information


Tables S1–S3.

## Data Availability

The data that support the findings of this study are not publicly available duo to ethical and privacy restrictions: consent forms do not allow data utilization by other research teams. The data may be available on request from the corresponding author and after additional and documented consent of parents involved in this study.
