# Supplementary material for: Does the brain sleep differently depending on intellectual abilities?
Source: CNS Neurosci Ther. 2023 Jul 23;30(2):e14378. doi: 10.1111/cns.14378 (PMC10848103; doi:10.1111/cns.14378)
Supplement: Supplementary file 1 — Tables S1–S3. [file CNS-30-e14378-s001.docx]

# Supplementary Materials

**Table S1. Questionnaire results for children with NIQ and HIQ**

|  | NIQ | N | HIQ | N | *p* |
| --- | --- | --- | --- | --- | --- |
| AESS, total | 4 (0-11) | 17 | 2 (0-21) | 21 | 0.43 |
| AESS pathological (> 10), n (%) | 2 (12) | 17 | 1 (5) | 21 | 0.58 |
| ISI, total | 4 (1-15) | 15 | 11 (0-18) | 20 | 0.08 |
| ISI pathological (> 10), n (%) | 3 (20) | 15 | 9 (45) | 20 | 0.16 |
| CDI, total | 11 (1-27) | 17 | 9 (0-36) | 21 | 0.62 |
| CDI pathological (> 16), n (%) | 6 (35) | 17 | 7 (33) | 21 | 1.00 |

All values in the columns 2 and 4 are reported as median (range) and n (%) when specified. The values in columns 3 and 5 indicate the sample size. The last column indicates p-value for comparisons between the two groups (NIQ vs HIQ) using Fisher’s Exact test or Wilcoxon test according to the results of the Shapiro-Wilk tests. NIQ: Normal intellectual quotient; HIQ: High intellectual quotient; AESS: Adapted Epworth sleepiness scale; ISI: Insomnia severity index; CDI: Child depression inventory.

**Table S2. Sleep macrostructure of children with NIQ and HIQ**

|  | NIQ | N | HIQ | N | *p* |
| --- | --- | --- | --- | --- | --- |
| TST, min | 532 (348-655) | 17 | 524 (349-623) | 24 | 0.49 |
| Sleep latency, min | 16 (7-61) | 17 | 28 (6-68) | 23 | 0.36 |
| REM latency, min | 135 (50-243) | 17 | 141 (56-209) | 24 | 0.96 |
| Sleep efficiency, % | 95 (83-98) | 17 | 96 (68-99) | 23 | 0.06 |
| N1, min | 58 (27-149) | 17 | 53 (23-95) | 24 | 0.16 |
| N1, % | 12 (5-27) | 17 | 10 (4-18) | 24 | 0.09 |
| N2, min | 253 (140-296) | 17 | 235 (137-333) | 24 | 0.36 |
| N2, % | 45 (33-57) | 17 | 46 (36-57) | 24 | 0.64 |
| N3, min | 113 (73-149) | 17 | 109 (65-146) | 24 | 0.54 |
| N3, % | 20 (13-35) | 17 | 21 (13-28) | 24 | 0.54 |
| REM, min | 105 (66-178) | 17 | 122 (64-225) | 24 | 0.12 |
| REM, % | 20 (15-32) | 17 | 23 (18-36) | 24 | 0.03 |
| WASO | 24 (9-88) | 17 | 21 (7-118) | 24 | 0.22 |
| Arousals, /h | 8.9 (4.8-14.8) | 17 | 10 (4.6-13.5) | 24 | 0.60 |
| Cycles, n | 5 (3-6) | 17 | 6 (4-6) | 24 | 0.40 |
| Cycle duration, mean | 107 (81-126) | 16 | 99 (79-131) | 23 | 0.25 |

All values in the columns 2 and 4 are reported as median (range). The values in columns 3 and 5 indicate the sample size. The last column indicates p-value for comparisons between the two groups (NIQ vs HIQ) using a t-test or Wilcoxon test according to the results of the Shapiro-Wilk tests. NIQ: Normal intellectual quotient; HIQ: High intellectual quotient; TST: Total Sleep Time; REM: Rapid Eye Movements; N1: Sleep stage 1; N2: Sleep stage 2; N3: Sleep stage 3; WASO: Wake After Sleep Onset

**Table S3. EEG spectral characteristics of children with HIQ and NIQ during NREM sleep**

|  | NIQ  N = 17 | HIQ  N = 24 | *p^unadjusted^* | *p* | η^2^ |
| --- | --- | --- | --- | --- | --- |
| Relative power |  |  |  |  |  |
| δ-slow | 53.1 (42-58.4) | 50.4 (45.1-58.5) | 0.50 | 0.45 | .020 |
| δ-fast | 11 (9.4-14.8) | 12.6 (9.4-14.9) | 0.16 | 0.25 | .098 |
| θ-slow | 5.1 (3.8-6.5) | 5.4 (4-7) | 0.32 | 0.21 | .117 |
| θ-fast | 2.8 (1.6-4.1) | 2.9 (1.8-7.3) | 0.42 | 0.44 | .032 |
| α-slow | 1.1 (0.7-1.9) | 1.1 (0.6-2.7) | 0.55 | 0.76 | .169 |
| α-fast | 1.4 (0.7-4.1) | 1.6 (0.7-5.4) | 0.64 | 0.52 | .163 |
| σ | 1 (0.5-2.9) | 1.3 (0.5-3.9) | 0.13 | 0.30 | .326 |
| β-slow | 0.5 (0.2-1) | 0.6 (0.3-1.5) | 0.11 | 0.18 | .185 |
| β-fast | 0.3 (0.1-0.6) | 0.3 (0.2-0.9) | 0.28 | 0.34 | .043 |
| γ | 0.1 (0-0.1) | 0.1 (0-0.3) | 0.77 | 0.89 | .025 |
| Absolute power |  |  |  |  |  |
| Mean |  |  |  |  |  |
| δ-slow | 1134 (445-2134) | 851 (414-2477) | 0.44 | 0.54 | .039 |
| δ-fast | 162 (69-243) | 156 (81-307) | 0.91 | 0.81 | .152 |
| Total |  |  |  |  |  |
| δ-slow | 856904 (355837-1705221) | 657690 (233097-1942273) | 0.25 | 0.26 | .040 |
| δ-fast | 136389 (63474-225966) | 120726 (45597-220116) | 0.43 | 0.43 | .051 |

All values in the columns 2 and 3 are reported as median (range), p-values are reported for unadjusted (*p-unadjusted*) and adjusted (*p*) models in column 4 and 5 respectively. Analyses were conducted on log-transformed values, η^2^ stands for the effect size of the model adjusted for age and sex (column 6). NIQ: Normal intellectual quotient; HIQ: High intellectual quotient; REM: rapid eye movement.
